# Alterations in the Gut Microbiome of Individuals With Tuberculosis of Different Disease States

**DOI:** 10.3389/fcimb.2022.836987

**Published:** 2022-03-29

**Authors:** Yue Wang, Yali Deng, Nianqiang Liu, Yanggui Chen, Yuandong Jiang, Zihao Teng, Zhi Ma, Yuxue Chang, Yang Xiang

**Affiliations:** ^1^ Department of Epidemiology and Biostatistics, College of Public Health, Xinjiang Medical University, Urumqi, China; ^2^ Department of Women and Children and Community Health, Xinjiang Production and Construction Corps Center for Disease Control and Prevention, Urumqi, China; ^3^ Department of Disease Control and Prevention, Xinjiang Production and Construction Corps Center for Disease Control and Prevention, Urumqi, China; ^4^ Centre for Tuberculosis and Leprosy Control and Prevention, Xinjiang Uygur Autonomous Region Center for Disease Control and Prevention, Urumqi, China; ^5^ Department of Tuberculosis Control and Prevention, Wulumuqi Center for Disease Control and Prevention, Urumqi, China

**Keywords:** tuberculosis, gut microbiota, 16S rRNA, Uyghur nationality, latent tuberculosis infection (LTBI)

## Abstract

**Objective:**

There is evidence that the gut microbiota play a regulatory role in the occurrence and progression of tuberculosis. The purpose of the current study was to explore the alterations in gut microbiome under different tuberculosis disease stages in the Uyghur population, clarify the composition of microbial taxonomy, search for microbial biomarkers and provide innovative ideas for individual immune prevention and for control strategies.

**Design:**

A case–control study of Uyghur individuals was performed using 56 cases of pulmonary tuberculosis (PTB), 36 cases of latent tuberculosis infection (LTBI) and 50 healthy controls (HC), from which stool samples were collected for 16S rRNA gene sequencing.

**Results:**

The results showed that the alpha diversity indexes of the PTB group were lower than those of the other two groups (P <0.001), while only observed species were different between LTBI and HC (P <0.05). Beta diversity showed differences among the three groups (P = 0.001). At the genus level, the relative abundance of *Bifidobacterium* and *Bacteroides* increased, while *Roseburia* and *Faecalibacterium* decreased in the PTB group, when compared with the other two groups, but the changes between the LTBI and HC groups were not significant. The classifier in the test set showed that the ability of the combined genus to distinguish between each two groups was 81.73, 87.26, and 86.88%, respectively, and the validation efficiency was higher than that of a single screened genus.

**Conclusion:**

The gut microbiota of PTB patients was significantly disordered compared with LTBI and HC, while the changes of LTBI and HC were not significant. In the future, gut microbiota could be used as a non-invasive biomarker to assess disease activity.

## Introduction

Tuberculosis (TB) is an ancient respiratory infectious disease caused by *Mycobacterium tuberculosis* (*Mtb*). It is a major global public health problem and a serious threat to human life and health. The World Health Organization (WHO) indicated that tuberculosis was the leading cause of death from a single infectious source and ranked the 13th leading cause of death worldwide. There were 9.87 million new cases worldwide in 2020, with an incidence rate of 127 per 100,000 (World Health Organization, 2021). Latent tuberculosis infection (LTBI) is defined as a consistent immune response to *Mtb* antigens without evidence of clinically evident active tuberculosis (Rajpal et al., 2020). The global LTBI case number is about two billion, of which 5 to 10% may develop active TB in their lifetime (Houben and Dodd, 2016; Colangeli et al., 2020), so the importance of the LTBI state as a large pool of potential patients must not be ignored (Chee et al., 2018). The occurrence and development of tuberculosis is influenced by many factors such as bacterial load and host resistance (Eribo et al., 2020), but TB can also evade the immune system for decades after exposure (Orme et al., 2015) and can circulate dynamically across a spectrum of diseases, depending on susceptibility and infection and progress to active TB (Petruccioli et al., 2016; Drain et al., 2018). Other epidemiological risk factors include HIV infection (Ford et al., 2015), malnutrition, air pollution (Lönnroth et al., 2010), smoking (Glickman and Schluger, 2016), and diabetes mellitus (Naidoo et al., 2019). The immunological basis of pathogenesis involves innate immunity and adaptive immunity, determined by the state of immune balance, with co-regulation by type 1 T-helper and Type 2 T-helper cells (Schwander and Dheda, 2011). However, the above factors cannot explain all the total population attributable risk percentage (PAR%) and the high incidence of morbidity and mortality, suggesting that there are still unconfirmed risk factors (Namasivayam et al., 2018). There is growing evidence that the microbiome might contribute to tuberculosis risk and disease. Through constant contact with the immune system, the microbiome can continuously participate in the immune response when there is a new infection (Gupta et al., 2018), so host microbial community has a regulatory effect on host immune response and *Mtb* survival mechanism (Osei Sekyere et al., 2020).

Many microbial communities exist in the human body, especially the gut microbiota, which is closely related to the immune system of the body (Young, 2017). The gut microbiome is directly involved in the development and function of peripheral immunity (Honda and Littman, 2016) and the gut–lung (Budden et al., 2017) axis is a recently discovered bidirectional axis affecting the correlation between intestinal flora, respiratory flora, and digestive and respiratory diseases. The gut microbiota and its metabolites can directly affect the growth of *Mtb* (Rooks and Garrett, 2016), as shown by the colonization of *Clostridium* in the intestinal tract of sterile mice with activated intestinal and systemic Treg cells and promoted IL-10 secretion (Mazmanian et al., 2005). IL-10 can inhibit the immunopathological damage caused by TB (Wong et al., 2020). In mice models with intestinal dysbacteriosis, *lactobacillus* or colonization with *Bacteroides fragilis* could induce changes in CD4^+^ T, Treg and other immune cells, affecting the growth of *Mtb* (Schirmer et al., 2016; Negi et al., 2019). Indole Propionic Acid (IPA), one of the metabolites of *Clostridium sporogenes*, can inhibit the proliferation of *Mtb* by enhancing the autophagy ability of macrophages, and is more effective against *Mtb* than anti-TB drugs (Kaufmann, 2018). Short-chain fatty acids (SCFAs) are the final products of dietary fiber fermentation by intestinal anaerobic bacteria, mainly including propionate and butyrate, which were associated with increased risk for active TB. SCFAs suppressed production of IFN-γ and IL-17A by both polyclonal- stimulated T cells and TB antigen stimulated peripheral blood mononuclear cells (PBMCs), resulting in inhibition of intracellular *Mtb* clearance (Segal et al., 2017). The specific gut microbiome is related to the immune status of the host and changes in the abundance of certain gut microbiota can also affect the outcome of resistance to invading *Mtb* by changing the level of immune cells. The composition of the gut microbiota varies depending on TB status; the findings of Winglee et al. showed that the mice infected with *Mtb*, the abundances of *Clostridium* and *Bacteroides* decreased (Winglee et al., 2014), and in the clinical trial conducted by Huang et al. it showed that the abundance of *Bifidobacterium* decreased in *Mtb*-infected patients (Huang et al., 2019). In active tuberculosis patients, there was an increased abundance of *Facobacterium*, *Rothia*, and *Eubacterium* (Maji et al., 2018). Another study found that when taking antibiotics for 6 months, the dominant intestinal flora such as *Rumenococcus*, *Lactobacillus*, and *Bacteroides* were greatly reduced (Wipperman et al., 2017). The above results were influenced by different geographical conditions, different types of populations and genetic characteristics, so there is no consistent conclusion for now.

Xinjiang is a multi-ethnic gathering area that has a high TB burden and Uygur, has the highest population proportion. Studies have shown that the Uyghur intestinal flora was different from other ethnic groups in healthy individuals (Zhang et al., 2015). Based on the regional and ethnic characteristics of Xinjiang, this study used 16S rRNA gene sequencing and analysis of TB and LTBI gut microbiome species and taxonomic composition to investigate the Uighur TB gut microbiome spectrum. The different species will reflect the intestinal microbial biomarkers under specific disease states and will provide new directions and targets for non-invasive diagnosis and intervention by improving the gut microbiome, to control the incidence of tuberculosis in the region.

## Materials and Methods

### Study Design and Sample Collection

This case–control study was conducted from January to July 2021 among Uighur PTB, LTBI and HC cases who were over 18 years old and were recruited from the region of Xinjiang, China, and had not taken antibiotics and other drugs that affected the gut microbiota (Falony et al., 2016; Zhernakova et al., 2016) for at least one month. Subjects excluded were those affected by HIV infection, diabetes, other lung diseases and other serious diseases, or those who were unwilling participants. The three groups of subjects were required to be comparable in gender, age and BMI.

All research subjects provided signed informed consent before sample collection and the protocol was approved by the Ethics Committee of First Affiliated Hospital of Xinjiang Medical University (20180223-159).

Fresh stool samples from the participants were collected using sterile containment and stored at −80°C immediately for further analysis.

### DNA Extraction and 16SrRNA Gene Amplicon Sequencing

Total genomic DNA samples were extracted using the QIAamp DNA Stool Mini kit (QIAGEN, Inc., Netherlands), following the instructions of the manufacturer and stored at −20 °C prior to analysis. PCR was used to amplify the hypervariable V3–V4 region of bacterial 16SrRNA genes with the forward primer, 338F: 5’-ACTCCTACGGGAGGCAGCA-3’ and the reverse primer, 806R: 5’-GGACTACHVGGGTWTCTAAT-3’. The libraries were constructed by for quality inspection. The qualified and quantified PE250 libraries were sequenced using the Illumina NovaSeq platform (Shanghai Personal Biotechnology Co., Ltd, Shanghai, China).

### Sequence Analysis

Microbiome bioinformatics were performed using Quantitative Insights Into Microbial Ecology (QIIME2 V.2019.4) (Bolyen et al., 2018). Raw sequence data were demultiplexed using the demux plugin followed by primer cutting with cutadapt plugin. Non-singleton amplicon sequence variants (ASVs) were generated after being quality filtered, denoised, merged, and chimeras were removed using the DADA2 plugin. Taxonomy was annotated with Greengenes database release 13.8 and classified at the taxonomic level of kingdom, phylum, class, order, family, genus, and species.

### Bioinformatic Analysis of 16SrRNA Sequencing

The 16SrRNA sequencing data analyses were performed using QIIME2 and R packages (v4.0.2). Alpha diversity indices were characterized by observed species, Pielou’s evenness, the Shannon and Simpson indexes. The Kruskal–Wallis H test was used to assess the differences between the three groups. Beta diversity was described by Nonmetric multidimensional scaling (NMDS), using UniFrac distance metrics and the Analysis of similarities (Anosim) was used to examine differences between groups. After singletons were removed, the feature table was counted to visualize the composition distribution of each sample at the phylum, class, order, family, genus, and species levels, and presented in a histogram. Linear discriminant analysis effect size (LEfSe) is a method of analyzing discrepancy, which directly analyzes the difference between all taxonomic levels simultaneously. The analysis used a cladogram showing the taxonomic hierarchy distribution of biomarker species of each group sample. Microbial potential functions were predicted by phylogenetic investigation of communities by the reconstruction of unobserved states (PICRUSt2) on KEGG databases to get predictive metabolic pathways, and conducted correlation analysis with different species demonstrated by heat maps. The sequence data of this study have been deposited in the GenBank Sequence Read Archive (SRA) of NCBI under the accession code BioProject PRJNA795263.

### Statistical Analysis

The Spss21.0 software and R Packages (V4.0.2) were used for data analysis. Individual characteristics of the interviewee were statistically described by the median and mean ± standard deviation (SDs) for continuous variables, while the Kruskal–Wallis test and analysis of variance (ANOVA) were used for inter-group differences. Frequency was used for categorical variables and the chi-square test was used for inter-group differences.

The Mann–Whitney U test was used for each two-group comparison of the top 50 genus level species relative abundance and species with differences among groups were selected out. The research object was divided into training and testing sets, and the random forest model was used to perform five repetitions of 10-fold cross validation and rank variables by importance based on previously screened differences variables, to find the optimal number of variables and their importance order. Filtered variables were used as predictors to draw receiving operating characteristic (ROC) curves, calculating the area under curve (AUC) and its 95% confidence interval (CI). The ROC curve of a single species of the combined species in each group was evaluated. Rank correlation was used to analyze the correlation between prediction pathways and different species. A P <0.05 was considered statistically significant.

## Results

### Sequence Composition Analysis

There were 56 PTB, 36 LTBI, and 50 HC cases included in this study. Age, gender, and BMI were comparable after statistical evaluation (P >0.05), as shown in **Table S1**.

**Figure 1:**
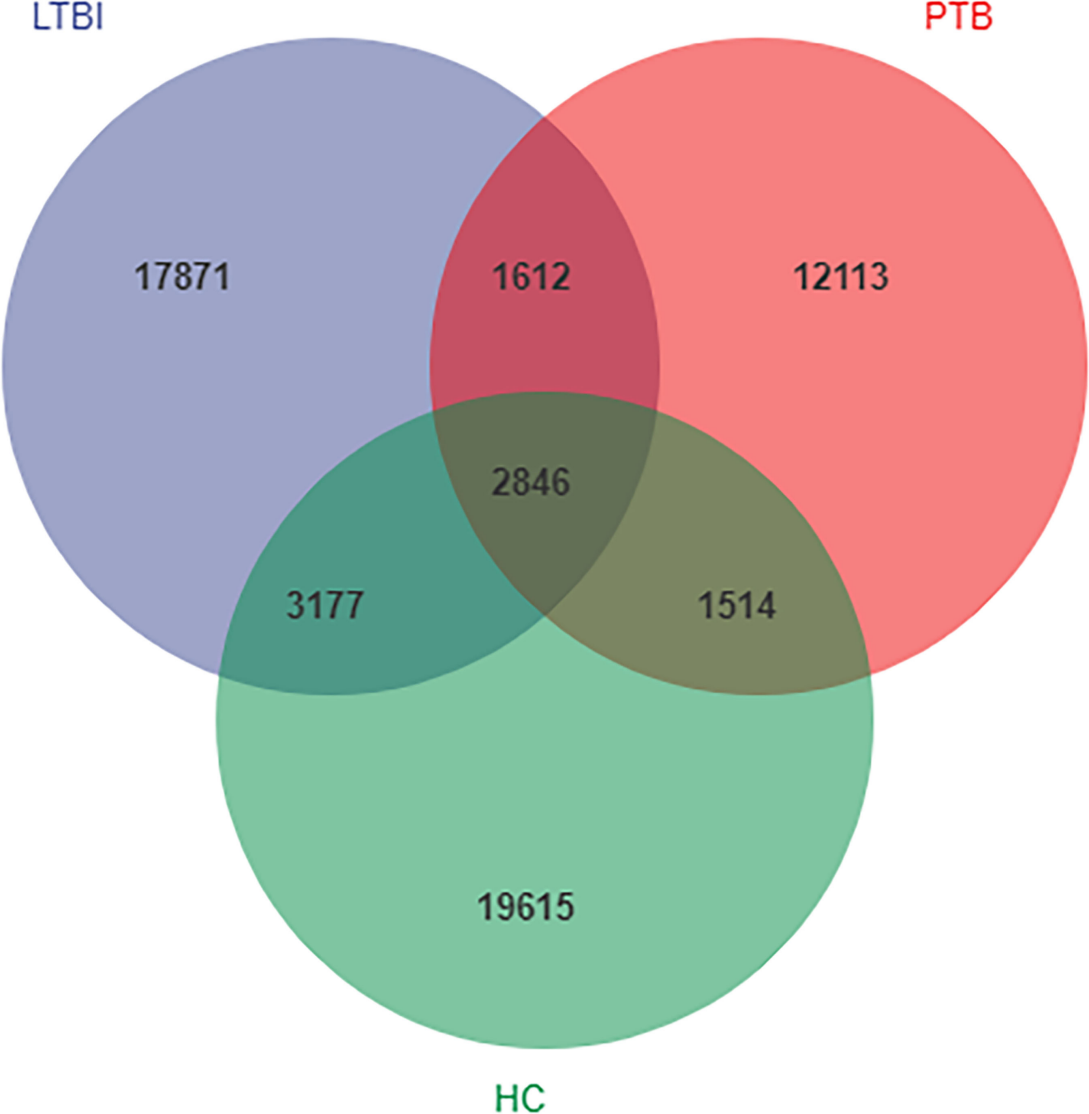
Venn diagram showing the shared and unique amplicon sequence variants (ASVs) in the flora of the three groups.

A total of 8,193,170 usable high-quality sequence reads were generated from 142 samples by quality filtering, denoising, merging and removing chimeras. These had an average of 57,698 sequences per sample, the sequence length ranged from 50 to 437 bp and the average sequence length was 413 bp. A total of 18,085 ASVs were obtained from the PBT group, 25,506 ASVs from the LBTI group and 27,152 ASVs from the HC groups. A total of 2,846 ASVs were shared among the three groups, where the PBT group contained 12,113 unique ASVs, the LBTI group contained 17,871unique ASVs and were compared to 19,615 unique ASVs from the HC group (**Figure 1**). The rarefaction curves of the three groups almost plateaued, indicating that the sequencing results were robust enough to reflect the diversity contained in the current samples (**Figure S1**).

### Alpha and Beta Diversity Analysis

**Figure 2:**
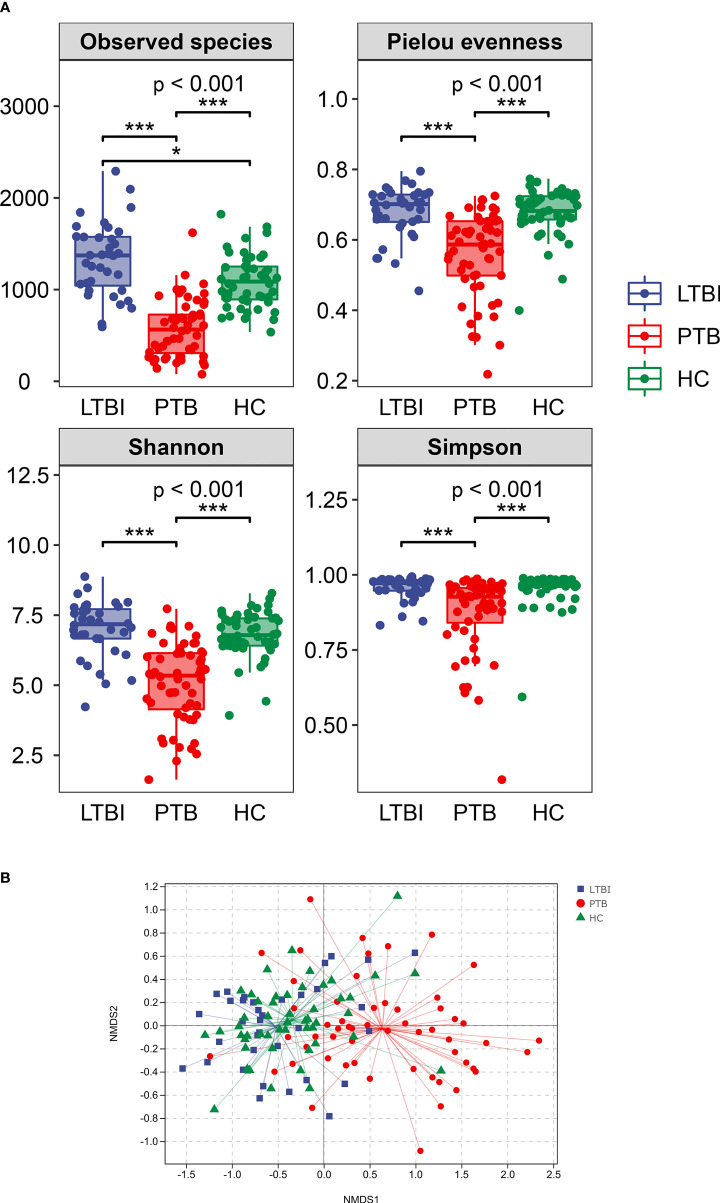
Comparison of the intestinal microbiota richness and diversity in three groups. **(A)** The alpha diversity was assessed using the above indexes. The P-values of the overall difference between groups obtained by the Kruskal–Wallis nonparametric test, markers of difference significance levels obtained after pairwise comparison of Dunn’s test between groups (*P < 0.05, ***P < 0.001). **(B)** NMDS represent beta diversity, measured by unweighted unifrac, the differences in the microbiome composition among groups were assessed by ANOSIM.

Alpha diversity was described using the observed species index, Pielou^’^s evenness index, and the Shannon and Simpson indexes. Overall, the Alpha diversity was decreased in the PTB group, when compared with LTBI and HC groups. The observed species were increased in the LTBI group compared with the HC group (P <0.001) (**Figure 2A**).

Beta diversity was demonstrated by Nonmetric Multidimensional scaling (NMDS) analysis, using UniFrac distance metrics, with the results in **Figure 2B** showing that there were significant differences among the three groups (P = 0.001).

### The Divergent Taxonomic Abundances and Composition of Microbiota Among Groups

According to the species taxonomic annotation, there were 28 microbial phyla, 88 microbial class, 160 microbial orders, 286 microbial families, 623 microbial genera, and 872 microbial species identified (**Table S2**).

**Figure 3:**
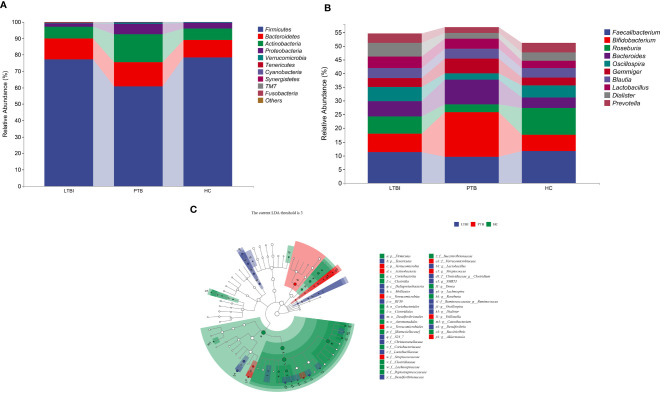
Taxonomic features of the fecal microbiota of patients in three groups. **(A)** The distribution of three groups at phylum level of top 10 species. **(B)** The distribution of three groups at genus level of top 10 species. **(C)** Cladogram is a taxonomic diagram showing the taxonomic hierarchy of the signified species in each group of samples by LEfSe.

At the phylum level, the composition of the top ten species is shown in **Figure 3A**, where the distribution of *Firmicutes*, *Verrucomicrobia*, *Tenericutes* and *Cyanobacteria* differed among groups. The abundance of *Firmicutes* and *Tenericutes* decreased in PTB, comparing with the LTBI and HC groups (**Figure S2**). At the genus level, *Bifidobacterium* was the most abundant genus at 16.35% in PTB, an increase of 59.20% compared with the LTBI group and 64.03% for the HC group. The most obvious reduction was *Roseburia* in PTB, compared with the LTBI and HC groups, the genus decreasing by 56.92 and 71.52%, respectively. The remaining changes are shown in **Figure 3B**.

The LEfSe analysis showed that there were 42 enriched species among the three groups. The *Actinobacteria* (class) was significantly enriched and had the highest abundance in the PTB group. In the LTBI group, *Dialister*, *Oscillospira*, and *Lactobacillus* were enriched at the genus level, while *Lactobacillaceae* was the highest accumulated at the family level. The phylum of *Firmicutes*, the class of *Clostridia*, the order of *Clostridiales*, the family of *Firmicutes* and the genus of *Roseburia* were significantly enriched in the HC group (**Figure 3C**).

### Gut Microbiome-Based Signature Distinguished Different TB States

The top 50 genera comprising about 80% of the total relative abundance were selected and compared between the three groups in pairs. Univariate analysis showed that 23 genera in the PTB and HC groups, 29 genera in the PTB and LTBI groups and four genera in the LTBI and HC groups had statistical differences. Random forest models were performed in the first two groups of training data sets and six out of 23 genera and four out of 29 genera were selected (**Figures S3**–**S6**). These indicators were used as predictor generated areas under the ROC.

**Figure 4:**
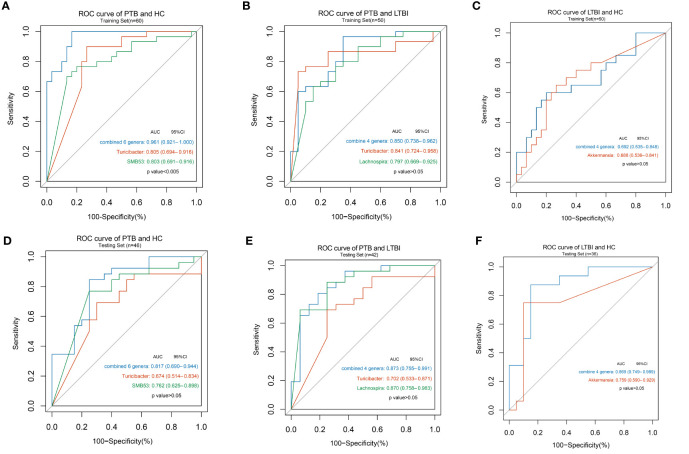
Disease states classification based on gut microbiome signature. Classification performance of random forest models by ROC for training set **(A)** in PTB and HC groups (n = 30 and 30); **(B)** in PTB and LTBI groups (n = 30 and 20); **(C)** in LTBI and HC groups (n = 20 and 30) and for testing set; **(D)** in PTB and HC groups (n = 26 and 20); **(E)** in PTB and LTBI groups (n = 26 and 16); **(F)** in LTBI and HC groups (n = 16 and 20).

In training sets, six genera, namely, *Weissella*, *Turicibacter*, *Lachnospira*, *Butyricicoccus*, *SMB53*, and *Veillonella* were combined and could distinguish PTB from HC with an AUC of 0.96 and a 95% CI of 0.92 to 1.00 (**Figure 4A**), four genera, namely, *Turicibacter*, *Lachnospira*, *Lactobacillus*, and *Actinomyces* could distinguish PTB from LTBI with an AUC of 0.85 and a 95% CI of 0.74 to 0.96 (**Figure 4B**), and four genera, namely, *Lactobacillus*, *Akkermansia*, *Lachnobacterium*, and *Bulleidia* could discriminate LTBI from HC with an AUC of 0.69 with a 95% CI of 0.54 to 0.85 (**Figure 4C**).

The classifying ability of the model was validated in an internal test data set. Using the six genera yielded an AUC of 0.82 with a 95% CI of 0.69 to 0.94 to discriminate PTB from HC (**Figure 4D**), using the four genera described above increased the AUC to 0.87 with a 95% CI of 0.75 to 0.99 to discriminate PTB from LTBI (**Figure 4E**) and using the four genera described above increased the AUC to 0.87 with a 95% CI of 0.75 to 0.99 to discriminate LTBI from HC (**Figure 4F**). The results of other single genus are shown in **Table S3** and ROC curves were also drawn.

### Prediction of Functional Potential

The results of 16 S rRNA genes sequencing were annotated in the KEGG database by PICRUSt2 to predict the sample functional abundance. A total of 32 level-II metabolic pathways were obtained in all samples.

**Figure 5:**
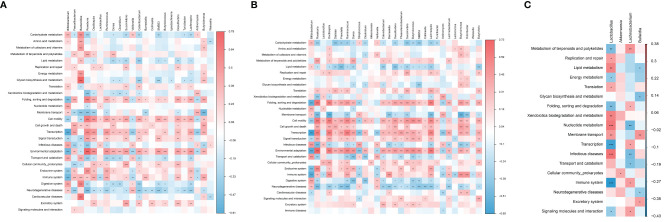
Correlation analysis between predictive metabolic pathways and different gut microbiota. The significant correlation **(A)** in PTB and HC groups; **(B)** in PTB and LTBI groups; **(C)** in LTBI and HC groups. The depth of the color in the heat maps signifies the strength of the correlation: red represents a positive correlation, whereas blue indicates a negative correlation. *P < 0.05, **P < 0.01, ***P < 0.001.

Correlation analysis was conducted between predicted metabolic pathways and different species, the results showed that *Bacteroides* was positively correlated with the pathway of Glycan biosynthesis and metabolism in PTB and HC group, *Roseburia* was positively correlated with the pathway of Environmental adaptation in PTB and LTBI group, and *Lactobacillus* was positively correlated with the pathway of Infectious diseases in the PTB and LTBI groups. The remaining significant correlations were detailed in heat maps (**Figures 5A–C**).

## Discussion

The immunomodulatory role of intestinal flora has been shown to be critical in the host response to tuberculosis, namely, the prevention of TB infection, reducing progression from latency, the disease severity, and the occurrence of drug resistance and co-infection (Hong et al., 2016). In order to explore the relationship between gut microbiota and tuberculosis in the Uyghur tuberculosis population in Xinjiang, three groups of subjects were recruited in the current study, to identify the microbiological composition and dominant species of each group based on 16SrRNA sequencing, to find microbial biomarkers under different disease states, predict the potential function of metabolic pathways, and to deduce the interactions between intestinal microbes and tuberculosis infection and incidence.

Hu et al. previously used 16Sr RNA sequencing to explore HC, TB, and LTBI populations, and the results showed that the alpha diversity in *Mtb* infected persons was slightly decreased, but there was no statistical difference observed between alpha and beta diversity (Hu et al., 2019). In another study, the species abundance and microbial diversity of PTB decreased compared with the healthy control group, and there were significant differences in the relative abundance of species between the two groups (Hu et al., 2019). However, Matthew et al. did not show any microbiological differences between LTBI and HC (Wipperman et al., 2017). The current study also showed a significant decrease in species richness and diversity in PTB compared with HC and LTBI, while HC and LTBI showed no difference in other alpha diversity indicators except the number of observed species. Beta diversity demonstrated significant differences in gut taxonomic composition, confirming that *Mtb* cases had intestinal microbiome disorders, which would be reflected by the reduction of microbial diversity.

At the phylum level, the human gut microbiota taxonomic composition is dominated by *Firmicutes* and *Bacteroidetes*, reduced levels of *Actinobacteria* and *Proteobacteria*, and also low abundance, but important, phyla like *Verrucomicrobia*, *Fusobacteria*, and *Euryarchaeota* (Becattini et al., 2016). In the current study, *Firmicutes*, *Bacteroidetes*, *Actinobacteria*, and *Proteobacteria* were also the microbiome with high relative abundances in the three groups of research cases, but *Firmicutes* (F) showed a significant downward trend in the PTB group compared with the other two groups and the relative abundance of *Bacteroidetes* (B) in PTB and LTBI increased slightly. The F/B ratio is related to the susceptibility of disease occurrence (Ley et al., 2006) and also possibly pro- and anti-inflammatory activity (An et al., 2014). In this study, the ratio of F/B decreased in the PTB and LTBI groups, which was consistent with the results of the study of Huang. The study of Huang further demonstrated that the gut F/B ratio was positively related to cytokine levels, such as pro-inflammatory cytokine IL‐1β and anti-inflammatory cytokine IL-4 (Huang et al., 2019). IL-1β eradicates invading pathogens by activating neutrophils and macrophages; IL-4 inhibits the activation of macrophages and interferes with the clearance of *Mtb* by Th1 cells, which is an important factor for promoting the occurrence of tuberculosis and the recurrence of chronic infection (Kulpraneet et al., 2019). In the study of *H. hepaticus*-colonized mice it was shown that the F/B ratio might also play a role in the higher inflammation and reduced immune inhibition of *Mtb*. In sum, increased *Bacteroidetes*, with a concomitant reduction in *Firmicutes* in the gut can cause dysbiosis and subsequent immune dysregulation that can ultimately affect the capacity of the immune system to defend the body against TB (Majlessi et al., 2017). *Actinobacteria* has been regarded as an important indicator phylum to distinguish TB from HC. It is considered to be a harmful bacterium phylum and the research by Luo showed that it had a higher abundance in recurrent tuberculosis patients than new PTB and HC (Luo et al., 2017). In this study, the relative abundance of *Actinobacteria* in PTB group was significantly higher than that in the other two groups, while LTBI was slightly higher than HC. The phylum *Tenericutes*, that contains many beneficial species, was diminished in the TB patient group (Lim et al., 2017; Khaliq et al., 2021) and in this study the relative abundance of this species in the PTB group was also lower than that in the other two groups.

At the genus level, this study showed that the relative abundance of *Bifidobacterium* in PTB was significantly higher than that of the other two groups, consistent with a case–control study of ATB and HC from Pakistan (Khaliq et al., 2021), but in contrast an Indian study showed enrichment in the HC group (Maji et al., 2018; Dhakan et al., 2019). *Bifidobacterium* is an opportunistic pathogen, causing bacteremia in patients that have immune deficiency and intestinal barrier impairment (Lokesh et al., 2018). The abundance of *Bifidobacterium* also decreased in patients who received anti-tuberculosis treatment (Wipperman et al., 2017; Hu et al., 2019), but increased in patients who completed treatment (Hu et al., 2019). *Roseburia* is reported to influence the production of SCFAs, namely, acetic, propionic and butyric acids (Estaki et al., 2016; Rivière et al., 2016) and these metabolites protect the body from pathogens and inflammation (Sun et al., 2017). In this study, *Roseburia* was enriched in the HC group, a result consistent with the study by Dhakan (Dhakan et al., 2019).

The ROC curves for the genus level were made to find biomarkers that could distinguish the three groups. The PTB and HC group in the training set of the combined index of discrimination was up to 96.11% and the identification ability for *Turicibacter* and *SMB53* were 80.5 and 80.3%, respectively. The discriminant ability of the combined index in the validation set was 81.7% lower than that in the training set and SMB53 had the highest discrimination of 76.2%. Hu et al. employed metagenomics and observed *Haemophilus parainfluenzae*, *Roseburia inulinivorans*, and *Roseburia hominis* species to distinguish PTB from HC, namely, the genera of *Haemophilus* and *Roseburia* (Hu et al., 2019). *Turicibacter* and *SMB53* (family *Clostridiaceae*) are both considered to exhibit pro-inflammatory property (Ye et al., 2018; Petersen et al., 2019) and a study of comparative analysis of the intestinal flora in type 2 diabetes and nondiabetic mice indicated that these two genera may be involved in the abnormal metabolism of type 2 diabetes (Horie et al., 2017). A review of the literature found no role for *Turicibacter* and *SMB53* in tuberculosis, so they would need to be further validated as potential microbial biomarkers. For the ability to identify PTB and LTBI group, the combined genera in training set was 85.0%, compared with that of *Turicibacter* and *Lachnospira* of 84.1 and 79.7% respectively. The identification ability of the combined indexes in the validation set was 87.3%. *Lachnospira* belongs to *Clostridiale* (order) and has 87.0% ability to discriminate PTB and LTBI, which is a single genus with high discriminatory ability. Luo et al. noted that *Lachnospira* decreased in abundance in patients with new and relapsing TB and was negatively correlated with CD4+T cell counts, which are important immune cells for TB (Luo et al., 2017). *Lachnospira* has also been shown to control airway inflammation in germ-free mice (Arrieta et al., 2015). The combining of the four indicators to distinguish between LTBI and HC was 86.9% in testing test, and *Akkermansia* which belongs to *Verrucomicrobiaceae* (family), had 75.9% ability to distinguish LTBI from HC. This genus currently contains only one member, the *Akkermansia Muciniphila* species (Ansaldo et al., 2019), and several studies have shown that Akkermansia Muciniphila is a new generation of probiotics, which is abundant in the gut microbiota of healthy individuals and exerts the effect of preventing and treating obesity, type 2 diabetes and other metabolic dysfunctions, suppressing inflammatory response and regulating the immune activity (Zhang et al., 2019). No studies on this microbial at the genus level have directly shown that it is associated with *Mtb* infection, but a microbiological study of patients with hepatitis showed that *Akkermansia* was lower abundant in patients with a higher degree of polymorphonuclear infiltration, a higher degree of histological inflammation, and its abundance was inversely correlated with the level of inflammatory response (Lang et al., 2020). We deduced the abundance of *Akkermansia* may have relationships with the different levels of tuberculosis infection, but this hypothesis needs to be further demonstrated. Considering the diagnostic effectiveness and easy identification, *Lachnospira* can distinguish LTBI and PTB on behalf of the combined four genera.

The above analysis focuses on the diversity of microflora, the composition of species, and the microflora biomarker. The functional potential of the microbiome needs to be a focus and PICRUSt2 is used and annotate 16S rRNA sequenced genes based on the KEGG database to predict their functional potential. The correlations between metabolic pathways and different species among the three groups were obtained, so there is a preliminary understanding of the microflora function in the tested samples and these results could provide guidance for the follow-up study of intestinal microbe function like metagenomics.

### Limitations

This research is based on a cross-sectional study and although the basic characteristics of the research objects are balanced and comparable among the three groups and have strict inclusion and exclusion criteria when collecting research objects, the individual differences are still unavoidable. Ideally, cohort studies can rigorously observe changes in the intestinal flora of a subject from health to infection to disease onset and subsequent treatment. Gut microbiota is affected by genetic and environmental factors such as diet, and there are many potential influencing factors among research objects, murine model should be considered in future experiments to minimize the possible influence. In addition, although the internal validation was built, the accuracy and universality of the model can only be further evaluated if the sample is expanded for external and independent verification. Xinjiang is a multi-ethnic region, only Uyghurs were selected as the research subjects and whether the current results can be extrapolated to other ethnic groups requires further verification. Finally, due to the shortcomings of the 16SrDNA sequencing technology used in the current study, species can only be annotated to the ‘genus’ level, mainly involving the composition of species and community diversity. Subsequent metagenomic studies can be conducted on this basis, to identify microorganisms down to the species or strain level, and conduct in-depth genetic and functional studies.

### Conclusions

The current study used 16S rRNA gene sequencing to explore the alterations in the gut microbiome of individuals with tuberculosis of different disease states, and find the intestinal microbial biomarkers which will provide new directions and targets for non-invasive diagnosis and intervention by improving the gut microbiome.

## Data Availability Statement

The datasets presented in this study can be found in online repositories. The name of the repository and accession number can be found below: SRA, NCBI; PRJNA795263.

## Ethics Statement

All research subjects provided signed informed consent before sample collection and the protocol was approved by the Ethics Committee of First Affiliated Hospital of Xinjiang Medical University (20180223-159). The patients/participants provided their written informed consent to participate in this study.

## Author Contributions

YW: Conceptualization, Formal analysis, Data curation, Visualization, Writing – original draft, Writing – review & editing. YD: Supervision, Data curation, Writing – original draft. NL: Conceptualization, Supervision, Data curation. YaC: Conceptualization, Data curation. YJ: Formal analysis, Visualization. ZT: Data curation, Formal analysis. ZM: Data curation, Formal analysis. Yu-C: Conceptualization, Formal analysis. YX: Conceptualization, Supervision, Data curation, Writing—original draft, Writing—review & editing. All authors listed have made a substantial, direct, and intellectual contribution to the work and approved it for publication.

## Funding

This study was funded by National Natural Science Foundation of China (No. 81860589). The funders had no role in the study design, data collection and analysis, decision to publish, or preparation of the manuscript.

## Conflict of Interest

Authors YW and YD are in Xinjiang Production and Construction Corps Center for Disease Control and Prevention (CDC) not a commercial affiliation.

The authors declare that the research was conducted in the absence of any commercial or financial relationships that could be construed as a potential conflict of interest.

## Publisher’s Note

All claims expressed in this article are solely those of the authors and do not necessarily represent those of their affiliated organizations, or those of the publisher, the editors and the reviewers. Any product that may be evaluated in this article, or claim that may be made by its manufacturer, is not guaranteed or endorsed by the publisher.

## Supplementary Material

Supplementary Table 1 Demographic characteristics of the cohorts.

Supplementary Table 2 Species taxonomic annotation at different levels.

Supplementary Table 3 Results of the ROC curve analyses of significant species in training and testing sets.

Supplementary Figure 1 The rarefaction curves of the three groups.

Supplementary Figure 2 Four species with differences in the top ten at phylum level.

Supplementary Figure 3 Number of optimal predictor variables in PTB and HC.

Supplementary Figure 4 Number ofoptimal predictor variables in PTB and LTBI.

Supplementary Figure 5 Rank the importance of the variables using random forest Mean Decrease in accuracy in PTB and HC.

Supplementary Figure 6 Rank the importance of the variables using random forest Mean Decrease in accuracy in PTB and LTBI.

## Abbreviations

PTB, pulmonary tuberculosis; LTBI, Latent tuberculosis infection; HC, Healthy controls; TB, Tuberculosis; Mtb, Mycobacterium tuberculosis; ATB, Active tuberculosis; IPA, Indole Propionic Acid; Anosim, Analysis of similarities; ASVs, Amplicon sequence variants; NMDS, Nonmetric multidimensional scaling; LEfSe, Linear discriminant analysis effect size; LDA, Linear discriminant analysis; ANOVA, Analysis of variance; ROC, Receiving operating characteristic curves; AUC, Area under curve; 95% CI, 95% confidence interval; F, *Firmicutes*; B, *Bacteroidetes*.
