# Supplementary figures and images for: Alterations in the Gut Microbiome of Individuals With Tuberculosis of Different Disease States
Source: Front Cell Infect Microbiol. 2022 Mar 29;12:836987. doi: 10.3389/fcimb.2022.836987 (PMC9001989; doi:10.3389/fcimb.2022.836987)

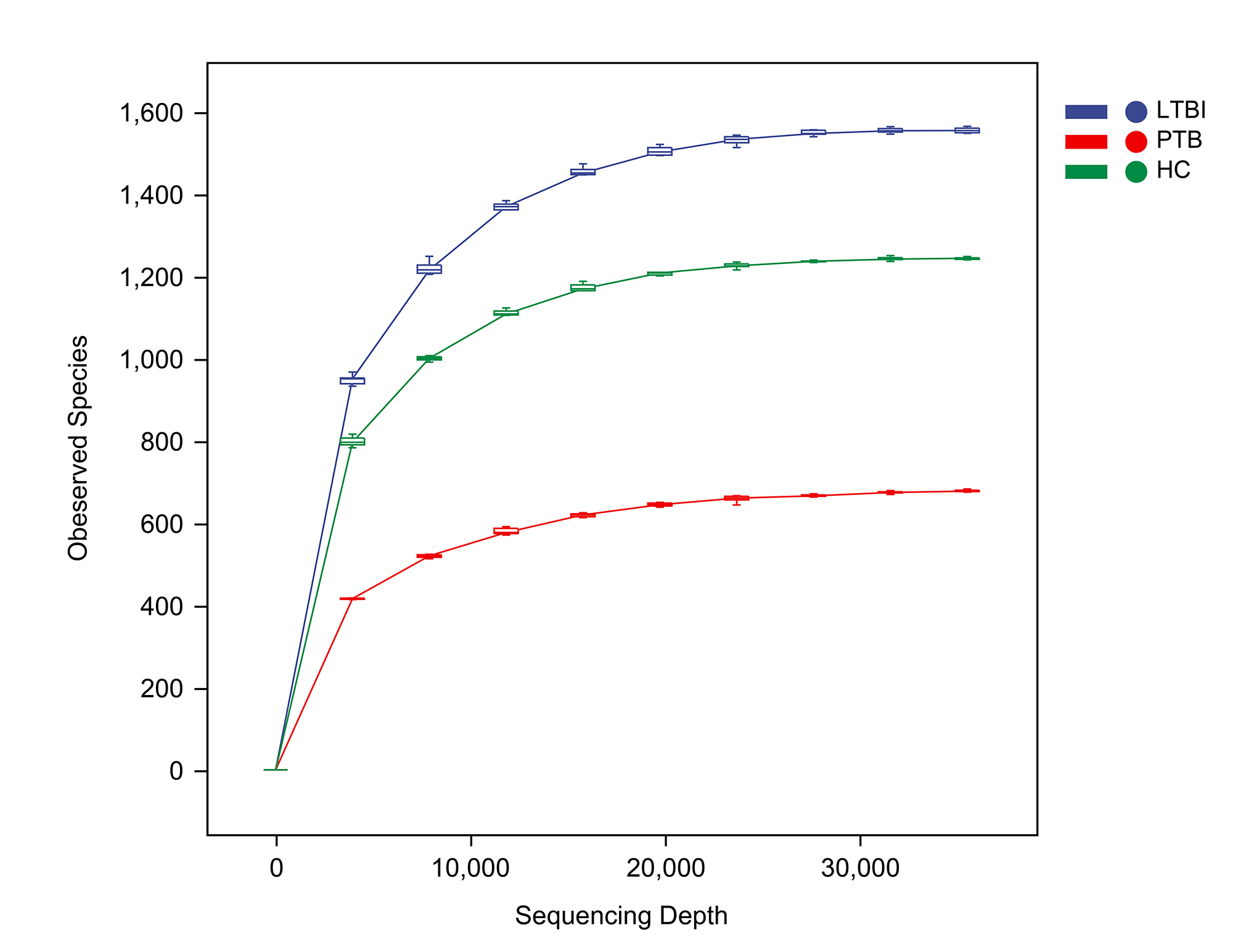

Supplement: Supplementary Figure 1 — The rarefaction curves of the three groups. [file Image_1.tif]

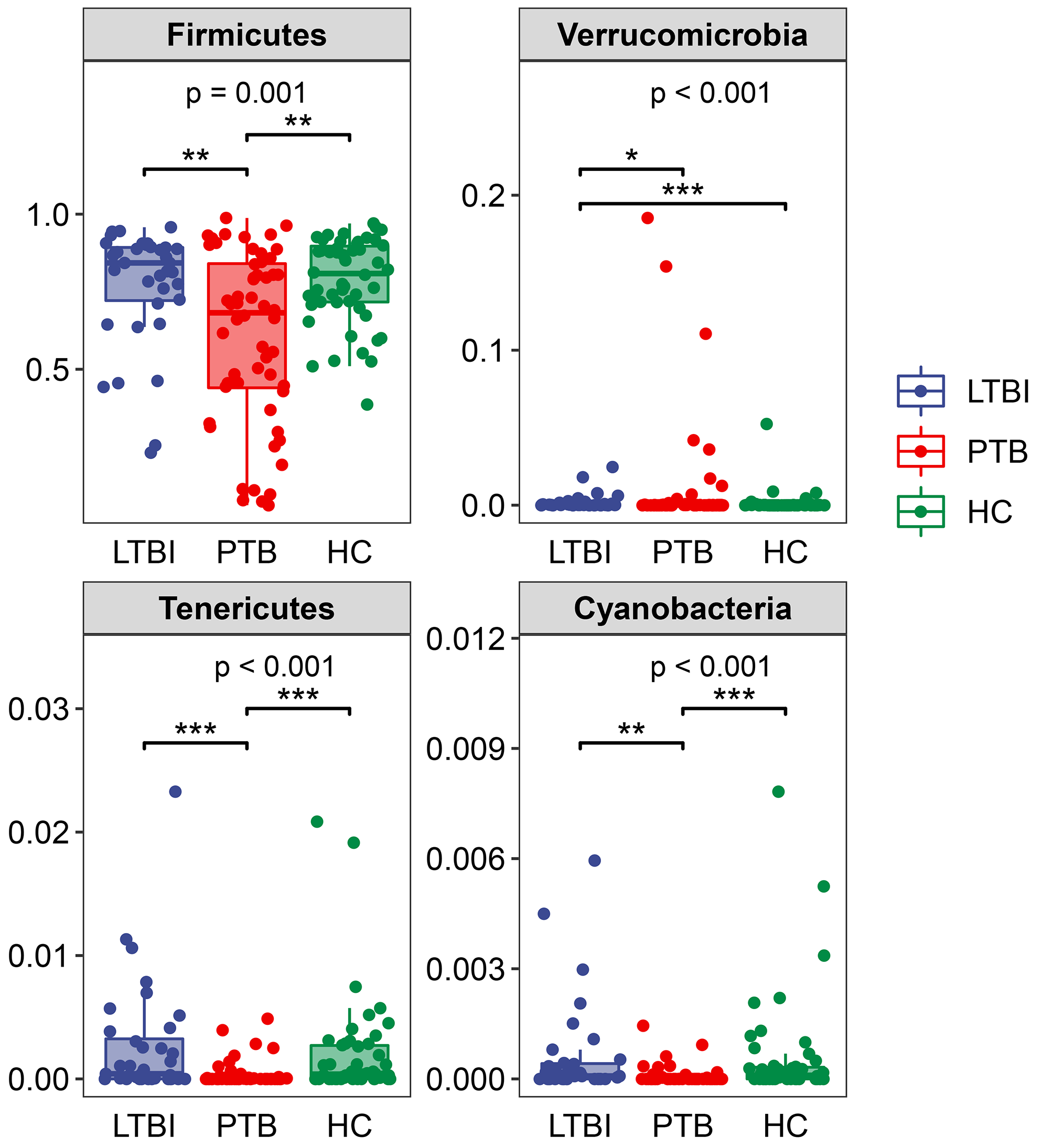

Supplement: Supplementary Figure 2 — Four species with differences in the top ten at phylum level. [file Image_2.tif]

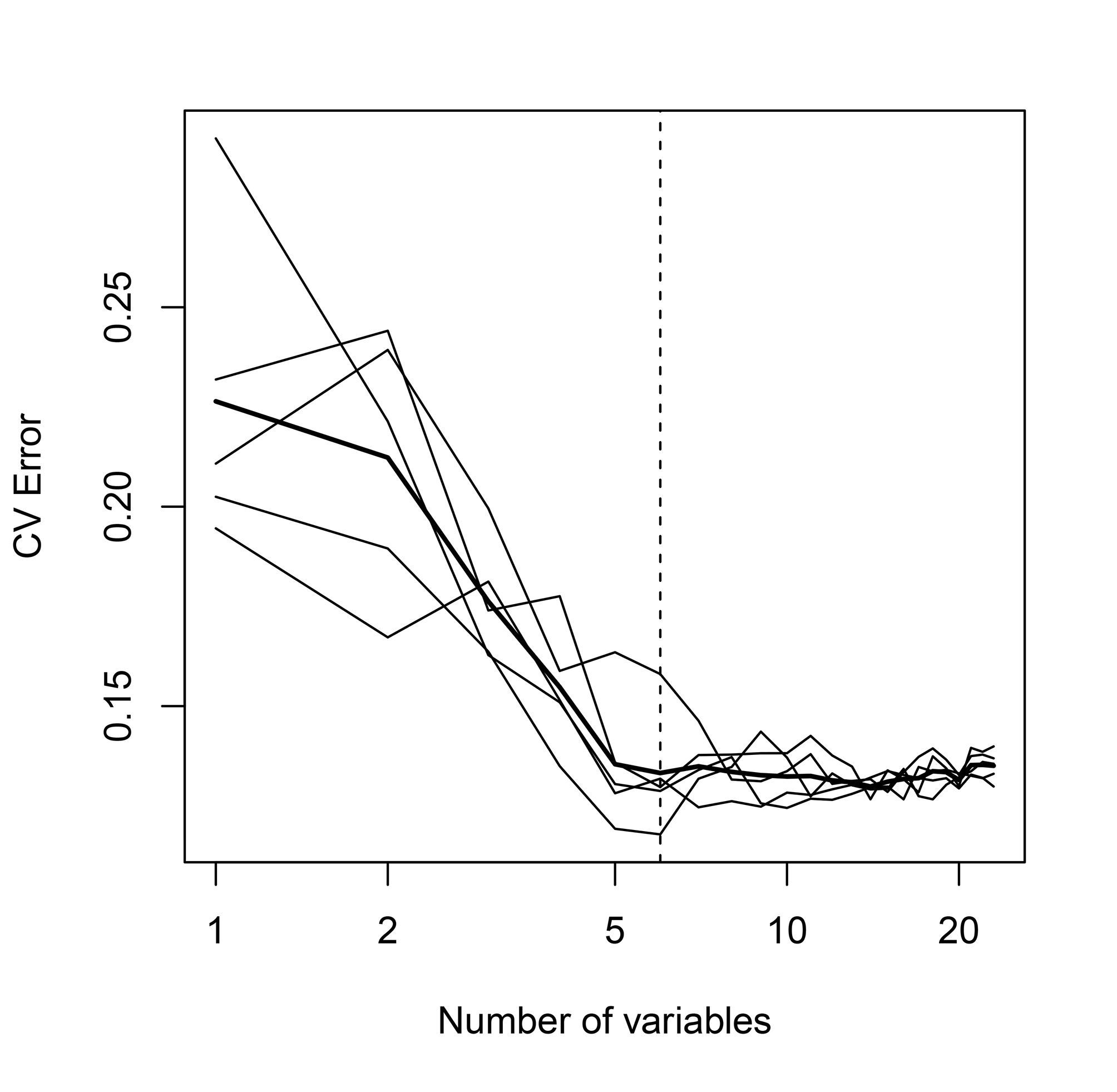

Supplement: Supplementary Figure 3 — Number of optimal predictor variables in PTB and HC. [file Image_3.tif]

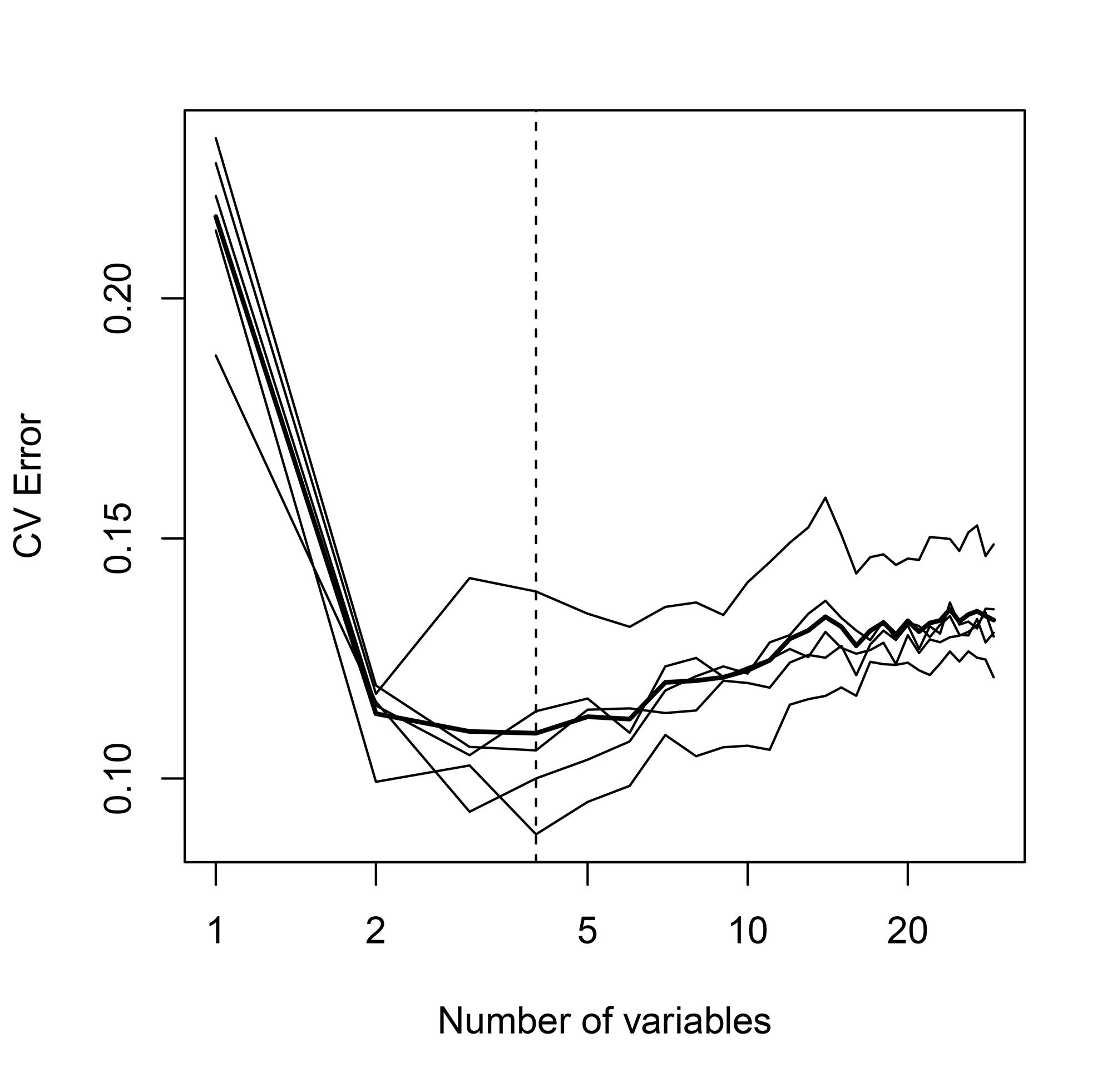

Supplement: Supplementary Figure 4 — Number ofoptimal predictor variables in PTB and LTBI. [file Image_4.tif]

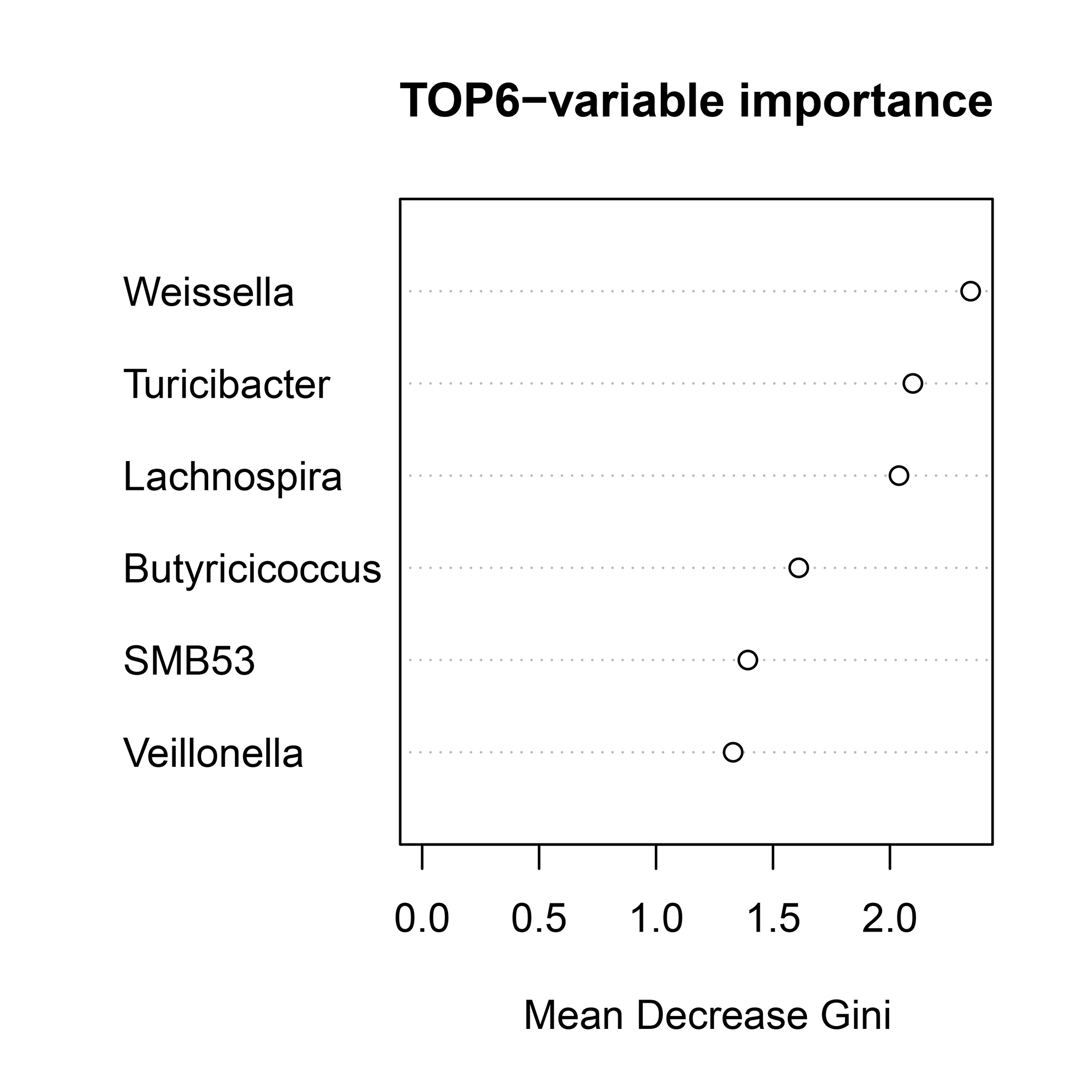

Supplement: Supplementary Figure 5 — Rank the importance of the variables using random forest Mean Decrease in accuracy in PTB and HC. [file Image_5.tif]

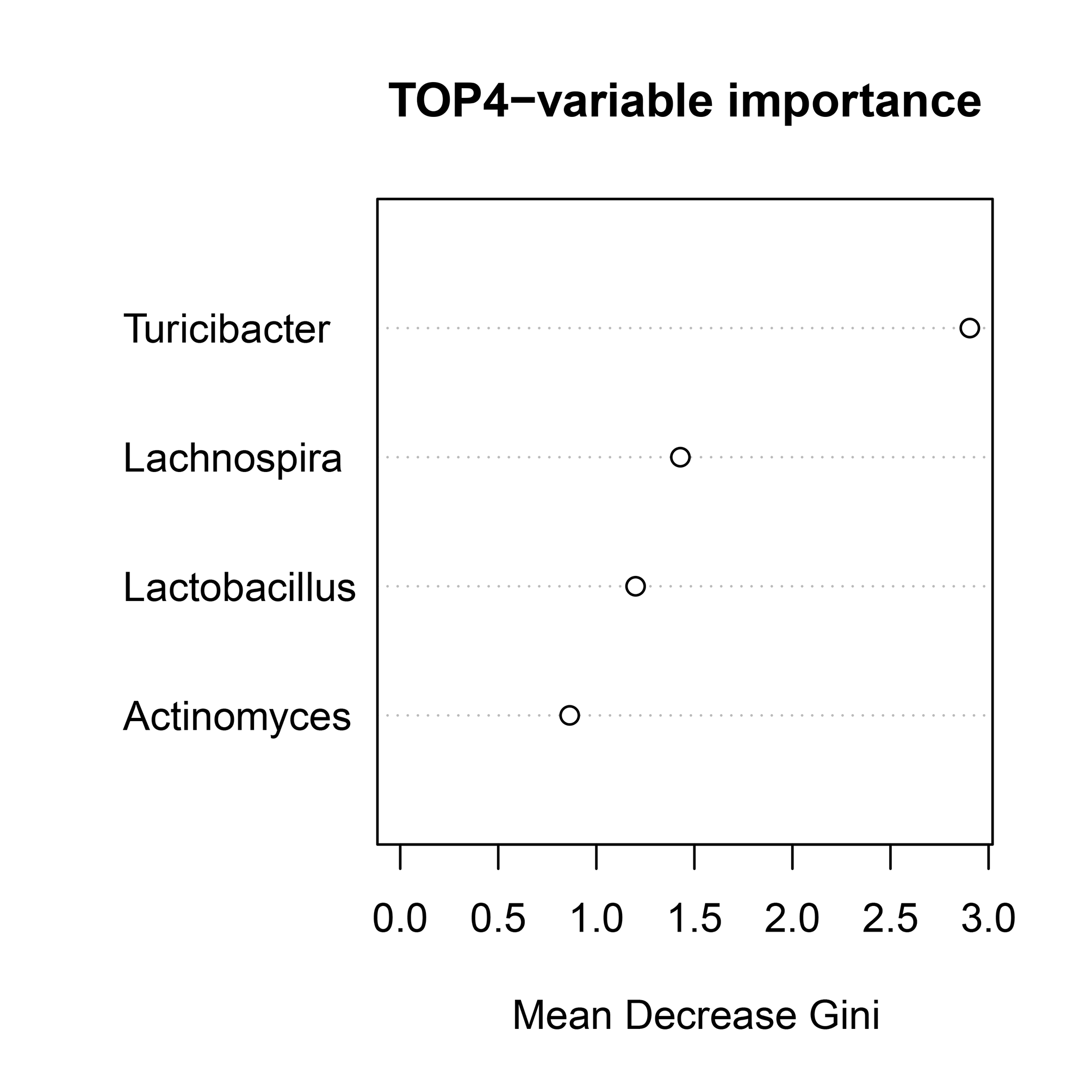

Supplement: Supplementary Figure 6 — Rank the importance of the variables using random forest Mean Decrease in accuracy in PTB and LTBI. [file Image_6.tif]
